# Supplementary material for: A hybrid achromatic metalens
Source: Nat Commun. 2020 Aug 4;11:3892. doi: 10.1038/s41467-020-17646-y (PMC7403425; doi:10.1038/s41467-020-17646-y)
Supplement: Supplementary file 1 — Supplementary Information [file 41467_2020_17646_MOESM1_ESM.pdf]

Supplementary information for “A Hybrid Achromatic Metalens”

F. Balli,[1,*] M. Sultan,[1,†] S. Lami,[1,‡] and J.T. Hastings[1,§]

[1] *University of Kentucky, Lexington, KY 40506, USA*

[*] fatih.balli@uky.edu

[†] m.sultan@uky.edu

[‡] sarah.lami@uky.edu

[§] todd.hastings@uky.edu

Supplementary Note 1: Control of Nanopillar Diameter

To optimize the multi-photon lithography process, we measured the diameter of the nanopillars as a function of laser power. We fabricated 11 different 10×10 nanopillar arrays with average powers ranging from $14 - 36$ mW. An example is shown in Supplementary Figure **1** which plots average diameter as a function of laser power. We fit a linear function to find the required laser power in order to obtain the target pillar diameter.

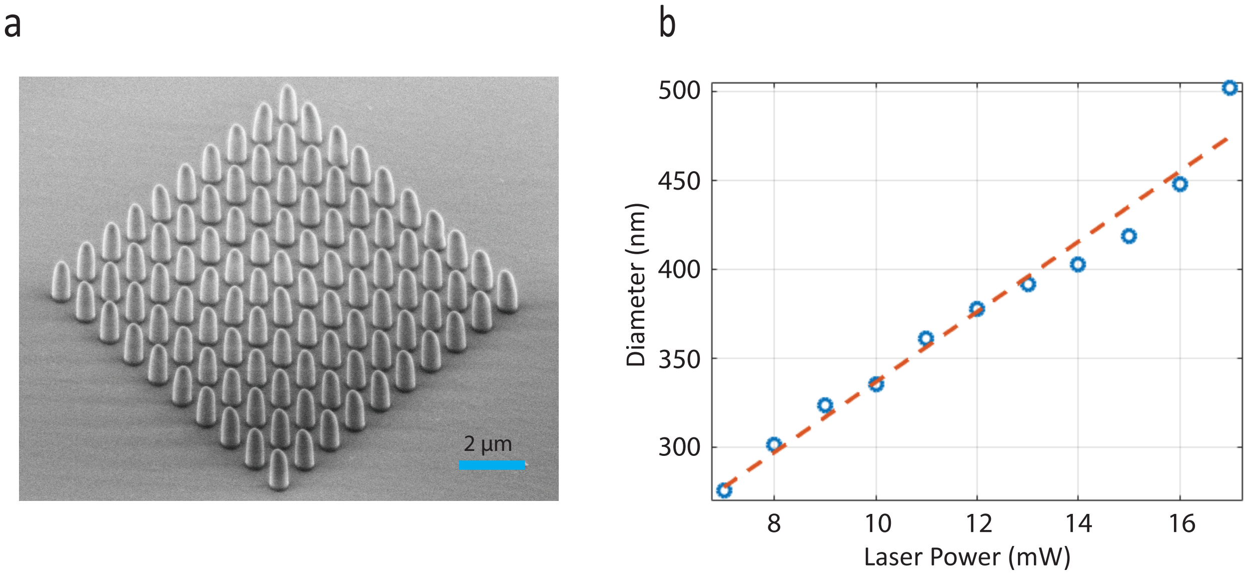

Supplementary Figure 1. Determination of pillar radius vs. laser power. **a,** SEM image of one of 11 fabricated nanopillar arrays. The laser power for this array was 13 mW. The height of the pillars is 1.5 μm. **b** Pillar diameter as a function of laser power with the linear fit shown. Source data are provided as a Source Data file for Supplementary Fig. 1b.

Supplementary Note 2: Comparison of Simulated and Experimental HAML Performance

In the main body of the paper we compared the hybrid metalens point-spread function (PSF) to the diffraction limited PSF. Focusing efficiency and focusing error were compared to other reported achromatic metalenses. However, the 20 μm EPD lens is small enough for the entire lens to be simulated using FDTD. Supplementary Figure **2** compares simulated vs. experimental results for each figure of merit. The simulated and experimental PSF match well (Supplementary Fig. **2**a,b) with a maximum deviation of the FWHM at $\lambda = 1300$ nm of 0.7 μm. The average focusing efficiency values are 61% (experimental) and 80% (simulated) as shown in Supplementary Fig. **2**b. Deviations are likely due to residual inaccuracies and

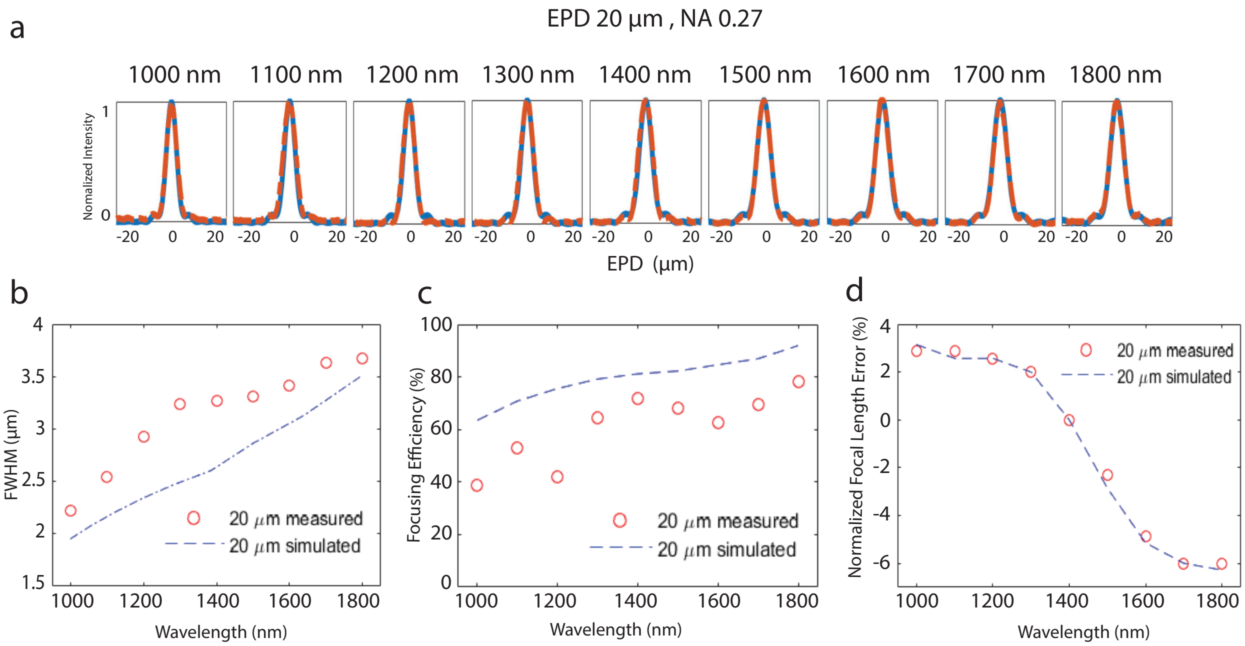

Supplementary Figure 2. Comparison of simulated and experimental results for the 20 μm EPD HAML. **a,** Simulated vs experimental focal plane intensity distributions. Red dashed line and blue continuous lines represent experimental and simulated data, respectively. **b,** Comparison of FWHM, focusing efficiency, and focusing error for experimental and simulated lenses. Source data are provided as a Source Data file for Supplementary Figs. 2b-d.

defects in the fabrication. The normalized focal length error shows excellent agreement between simulated and experimental results as well (see Supplementary Fig. **2**c).

Supplementary Note 3: Exploring the 3D Design Space

The ray tracing method provides us different lens powers for different separation distances between the TEs. Higher NA values can be obtained by increasing the amount of separation between TEs. However, this results in rotation of the phase derivative curve with respect to the origin. In order to well-approximate the trend, we fit an ellipse function to the phase derivative curve as a function of radial coordinate and rotation angle. The integration with respect to radial coordinate gives

$$\phi_{\pm}(r,\psi) = \frac{\pm\text{EPD}\phi'_o}{2}\arctan\sqrt{\frac{8r^2}{\text{EPD}^2 + 4\phi_o'^2 - 8r^2 + (\text{EPD}^2 - 4\phi_o'^2)\cos(2\psi)}} + \frac{r}{2}\phi'(r,\psi). \tag{S1}$$

Figure 2e shows an example of non-zero rotation angle. In our plot, we set the parameters as EPD=$22.6, 20$ μm, $\phi'_o = 0.39, 0.44$ $rad/\mu m$ and $\psi = 1.2°, 1.5°$ for the first and second

TEs, respectively.

We define the separation as the distance between the maximum height of the first TE and bottom layer of the second TE. We limit the maximum separation between the TEs to 7 μm due to fabrication limitations and low focusing efficiency values beyond this limit. However, the finite size of the TEs should be considered in the ray tracing algorithm to obtain the correct target phase shift. In our design, the correction term is added to the separation distances as 1 and 3.5 μm for the merged and air-spaced HAML structures, respectively.

To explore the design space, we fabricated two sets of lenses with same aperture size (40 μm) and different focal lengths (NA = 0.06, 0.32). The effective separation distance between the lenses are chosen as 1 μm and 10 μm for NA=0.06 and 0.32 lenses, respectively. We simulate and experimentally verify that additional separation between the lenses results in higher NA values compared to merged HAML as shown in Fig. 3c. In order to explore the effect of separation distance on performance, we sweep the distance for a fixed aperture size HAML. We choose EPD and separation range as 20 μm and $2-7$ μm. The longer separation allows us to reach higher NA values. Supplementary Fig. **3**a shows the trend of NA for different distances. While a decrease in focusing efficiency is noted, shown in Supplementary

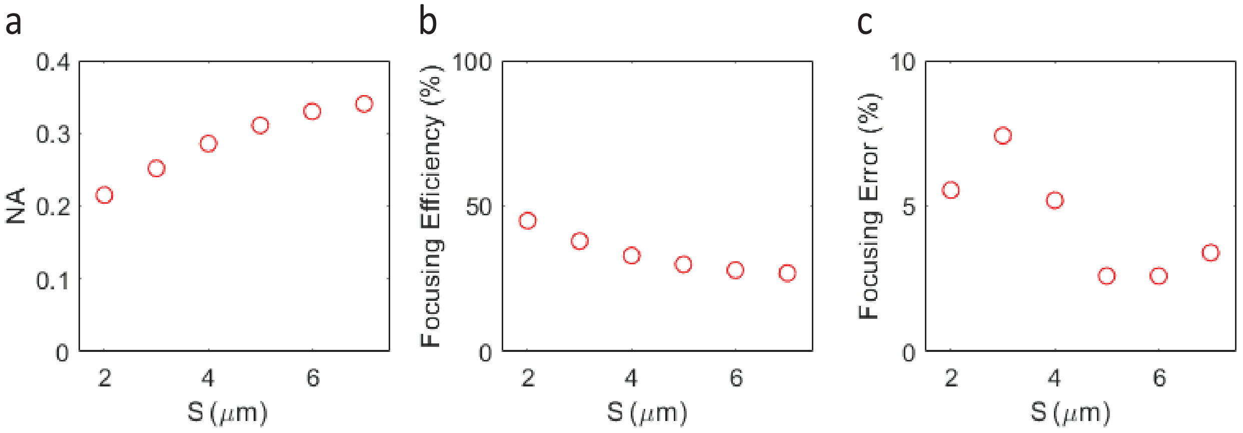

Supplementary Figure 3. Simulation results for the 20 μm EPD air-spaced HAML for various separation distances, S, between the elements. **a,** Highest NA within the region of convergence for a given separation distance. **b,c** Simulated average focusing efficiency and average focal length error as a function of separation distance. The average is taken by 9 different wavelengths from 1000 to 1800 nm with 100 nm step size. Source data are provided as a Source Data file for Supplementary Figs. 3a-c.

Fig. **3**b, the air-spaced HAML remains achromatic as shown in Supplementary Fig. **3**c.

In order to test the validity of Eq. S1 for larger aperture sizes, we fabricated a 100 μm EPD and 0.24 NA air-spaced HAML with 7 μm separation. Although diffraction-limited performance is achieved, a drop in focusing efficiency is noted. Supplementary Fig. **4**b shows the focusing efficiency values for a set of discrete wavelength range. The average focusing efficiency is 19% which is considerably lower than the merged HAML performance.

Supplementary Note 4: Dispersion Characteristics of Nanopillars

Two geometrical variables of the nanopillar structure allow us to choose different height and radius pairs that satisfy the target phase shift at a specific radial coordinate. Among the sets, we choose the pair which minimizes the penalty function $|e^{i\phi(R)} - T(h,d)e^{i\phi(h,d)}|$ where $T(h,d)e^{i\phi(h,d)}$ is the complex transmission as a function of height and diameter of the nanopillars. An example plot of phase is provided in Fig. 3a of the main text, and Supplementary Fig. **5**a provides an example plot of transmission at λ_{min} in terms of the geometrical variables. Alternatively, one could consider a penalty function that minimizes the phase error of a HAML structure across the entire wavelength range of operation. This optimization would exploit the geometrical dependence of group delay and group delay dispersion, as illustrated in Supplementary Fig. **5** b and c. Such an optimization may

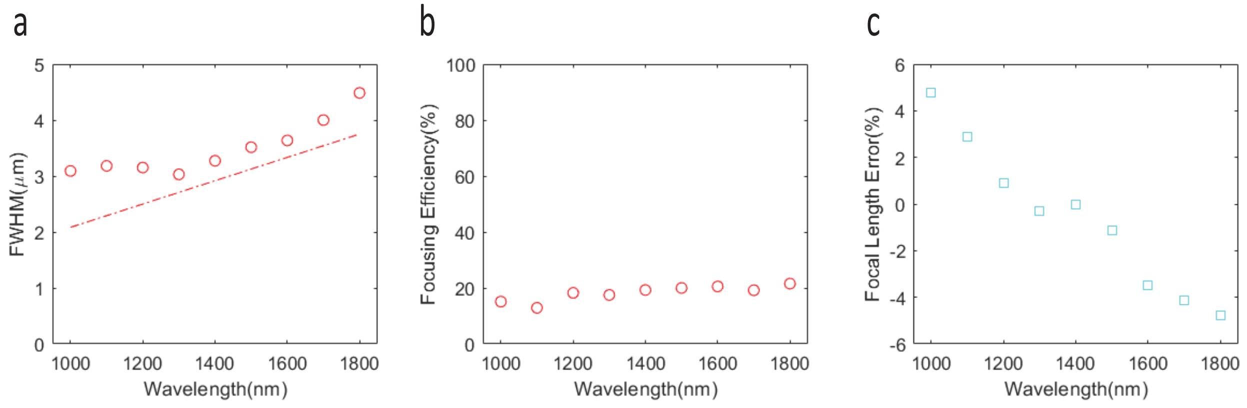

Supplementary Figure 4. Experimental results for the 100 μm EPD, 0.24 NA air-spaced HAML. **a,** Measured FWHM at focal plane. Dashed and continuous lines represent experimental and diffraction-limited data, respectively. **b,** Measured focusing efficiency values. **c,** Normalized focal length error as the function of wavelength. Source data are provided as a Source Data file for Supplementary Figs. 4a-c.

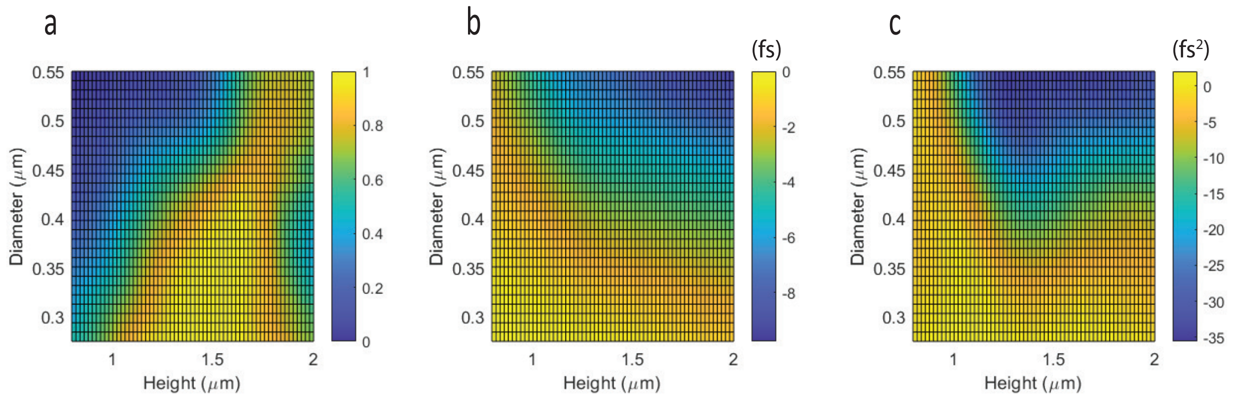

Supplementary Figure 5. Transmission and dispersion characteristics of the nanopillar structure **a,** Absolute transmission of the nanopillars at λ_{min} for different height and diameter values **b-c**, Group delay and group delay dispersion of the nanopillars.

further reduce the focal length error, but such an improvement is likely to come at the expense of focusing efficiency as transmission would necessarily receive reduced weight in the penalty function. Thus, we expect optimization of HAMLs with more complex, and application dependent, penalty functions to be an important subject for future work.

Supplementary Note 5: Optical Testing Apparatus

The apparatus for characterization of the metalenses is shown in Supplementary Fig. **6**. The key components are a supercontinuum source, a motorized translation stage, an objective lens, a tube lens, and a broadband (VIS-NIR) camera.

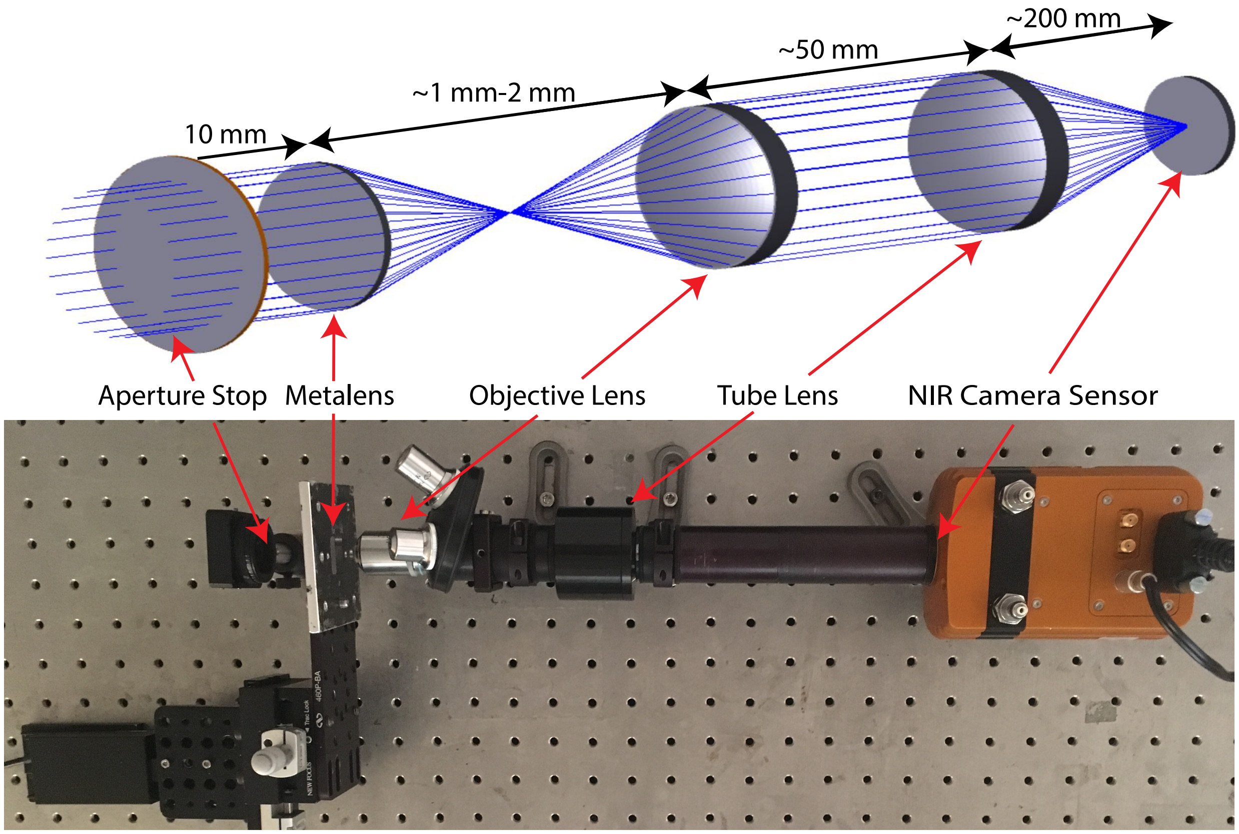

Supplementary Figure 6. Optical testing setup with approximate distance between components. Distance between the metalens and objective lens is variable and controlled by the motorized translation stage. The incident light emerges from the supercontinuum light source (not shown). For imaging, and the objective and tube lens relay the image formed by metalens to the focal plane array of the NIR camera with approximate 45X magnification.
